# Towards evenly distributed grazing patterns: including social context in sheep management strategies

**DOI:** 10.7717/peerj.2152

**Published:** 2016-06-21

**Authors:** Agustina di Virgilio, Juan Manuel Morales

**Affiliations:** Grupo de Ecolgía Cuantitativa, INIBIOMA, CONICET-UnComa, Bariloche, Río Negro, Argentina

**Keywords:** Selective grazing, Resource selection, Rangelands, Heterogeneous mixed flocks, Social rank, Sustainable management strategies, Overgrazing

## Abstract

**Background.** A large proportion of natural grasslands around the world is exposed to overgrazing resulting in land degradation and biodiversity loss. Although there is an increasing effort in the promotion of sustainable livestock management, rangeland degradation still occurs because animals’ foraging behaviour is highly selective at different spatial scales. The assessment of the ecological mechanisms modulating the spatial distribution of grazing and how to control it has critical implications for long term conservation of resources and the sustainability of livestock production. Considering the relevance of social interactions on animals’ space use patterns, our aim was to explore the potential effects of including animals’ social context into management strategies using domestic sheep grazing in rangelands as case study.

**Methods.** We used GPS data from 19 Merino sheep (approximately 10% of the flock) grazing on three different paddocks (with sizes from 80 to 1000 Ha) during a year, to estimate resource selection functions of sheep grazing in flocks of different levels of heterogeneity. We assessed the effects of sheep class (i.e., ewes, wethers, and hoggets), age, body condition and time since release on habitat selection patterns.

**Results.** We found that social rank was reflected on sheep habitat use, where dominant individuals (i.e., reproductive females) used more intensively the most preferred areas and low-ranked (i.e., yearlings) used less preferred areas. Our results showed that when sheep grazed on more heterogeneous flocks, grazing patterns were more evenly distributed at all the paddocks considered in this study. On the other hand, when high-ranked individuals were removed from the flock, low-ranked sheep shifted their selection patterns by increasing the use of the most preferred areas and strongly avoided to use less preferred sites (i.e., a highly selective grazing behaviour).

**Discussion.** Although homogenization and segregation of flocks by classes are common practices to increase flock productivity, we are proposing an alternative that employs behavioural interactions in heterogeneous flocks to generate more evenly distributed grazing patterns. This practice can be combined with other practices such as rotational grazing and guardian dogs (to decrease mortality levels that may be generated by sheep grazing on more risky habitats). This does not imply any modifications of livestock stocking rates and densities or any additional investments for labour and materials. Considering livestock behaviour is critical for the design of sustainable management practices that balance landscape conservation and livestock productivity.

## Introduction

A large fraction of natural grasslands around the world is exposed to overgrazing and high stocking rates, leading to land degradation and biodiversity loss of an environment that besides holding key natural resources sustains socio-economically a vast proportion of human population (MA, 2006; FAO, 2009; Sayre et al., 2013). Even though there is an increasing effort in the promotion of sustainable range management (Morghan, Sheley & Svejcar, 2006; Briske et al., 2008; Boyd & Svejcar, 2009), rangeland degradation still occurs because grazing behaviour is highly selective at different spatial scales (Senft et al., 1987; Bailey et al., 1996). These heterogeneous patterns of space use often produce overgrazing at the most preferred areas while other areas remain under-grazed (Bailey, Dumont & WallisDevries, 1998; Pringle & Landsberg, 2004). Although herbivores’ movement decisions are related to the distribution of resources (e.g., Fortin, Fryxell & Pilote, 2002; Fryxell, Wilmshurst & Sinclair, 2004), for animals living in groups, social context can also be an important factor influencing their movement and patterns of space use (Scott, Provenza & Banner, 1995; Scott, Banner & Provenza, 1996; Couzin et al., 2005; Haydon et al., 2008). As a consequence, the movement patterns of gregarious herbivores can result in socially-generated disturbances that are somehow independent of resource distributions (Bailey et al., 2001; Seabloom & Reichman, 2001; Bailey, 2004).

The social environment of group-living herbivores has several benefits on individual fitness that are related to knowledge transmission from conspecifics’ experiences (Scott, Provenza & Banner, 1995; e.g., Scott, Banner & Provenza, 1996) and minimization of predation risk (Pinchak et al., 1991; Plumb & Dodd, 1993; Etzenhouser et al., 1998; Fortin et al., 2003; Fortin et al., 2005; Frair et al., 2005; Rubio et al., 2008; Fortin et al., 2009). Nevertheless, when resources are scarce and heterogeneously distributed across the landscape, animals have to balance the benefits of group living with the competition for resources (Childress & Lung, 2003; Veiberg et al., 2004; Fortin et al., 2009). This trade-off may contribute to the development of hierarchical structures, in which animals are part of a social rank that could determine their grazing and movement patterns (Thouless, 1990; Veiberg et al., 2004). Regardless of which factors determine an animal’s position in a social rank, once the hierarchy is established, higher-rank individuals have priority access to preferred areas while lower-rank individuals use less preferred areas in the landscape which otherwise would be underused (Petocz, 1973; Bøe, Berg & Andersen, 2006).

Our general aim was to explore the potential effects of including the animals’ social context into management strategies using domestic sheep (*Ovis aries*, Linnaeus 1758) grazing in Patagonian rangelands as case study. In particular, we wanted to explore which factors influenced the development of a social rank when sheep graze in heterogeneous flocks and if this social structure favours the development of more evenly distributed grazing patterns. Considering that animals could show different movement behaviours according to their age (e.g., Favre, Martin & Festa-Bianchet, 2008), sex (e.g., Michelena et al., 2005), nutritional condition and their energy requirements (e.g., Vervaecke, Roden & De Vries, 2005) we assessed their influence on sheep selection patterns.

## Material & Methods

### Study area

Empirical data on sheep movement, body condition, and landscape traits were collected at *Fortín Chacabuco* Ranch (Supplemental Information 1). This farm is located in Los Lagos department (NW corner at 40°57′04.58″S, 71°08′44.14″W; SW corner at 40°59′45.27″S, 71°12′10.21″W; NE corner at 40°59′17.54″S, 71°05′52.75″W; SE corner at 41°03′09.17″S, 71°08′16.45″W) and belongs to the Pre-mountain range ecological area of Argentinean Patagonia (Cabrera, 1976). The landscape is characterized by mountain chains and hills, crossed by several rivers and water streams. The weather is cold, with mean annual temperatures of 10 °C and annual precipitations that range from 300 to 700 mm, concentrated during the cold season (i.e., May–August). Vegetation corresponds to the Sub-Andean district (León et al., 1998), composed by grasslands (dominated by *Stipa speciosa var major* in lower lands and by *Festuca pallescens* in upper lands), and wetlands of different extensions. Smaller wetlands are frequently associated to native and riparian forests.This ranch is characterized by high heterogeneity in sheep grazing patterns, with some areas intensively used and with degradation signs, and others under-used with high levels of litter accumulation from un-grazed senescent plants that decreases their productivity (Paramidani, Doffigny & Codesal, 2014).

### Data collection and spatial data processing

#### Landscape data

In order to characterize the landscape where sheep grazed, we mapped the vegetation units of each paddock were livestock was kept during the sampling period. First, we identified and defined polygons of landscape units that we were able to classify using Google Earth imagery (Digital Globe Quickbird. Resolution: 15 m per pixel. 10-16-2013. Accessed: September 2014: Supplemental Information 1, Fig. 1); then we corroborated and defined each polygon’s limits by matching them with an unsupervised classification from a Landsat TM (2011) satellite image using ArcGIS (ESRI 2011. ArcGIS Desktop10.1. Redlands, Environmental Systems Research Institute). This classification was then validated through vegetation sampling that consisted in placing transects inside each polygon, in which we located a 1 m^2^ plot every 100 m. These points were selected previous to fieldwork on a Google Earth file containing the polygons of vegetation units. The number of transects depended on the size of each polygon (i.e., in wider polygons we placed two or more transects), and the length depended on the polygon’s length. In each plot we registered every plant species present, their percentage of cover, and the percentage of cover of litter and bare ground. The species were recognized in the field, and we took samples only from those specimens that could not be identified in the field (permit number 1339). By using a vegetation guides for this region (e.g., Bonvissuto & Somlo, 1995) we were able to identify eight main landscape units: central wetland, peripheral wetland, grassland, shrubland-grassland, native forest (dominated by *Maytenus boaria*, *Austrocedrus chilensis*, and *Nothofagus* spp.), riparian forest (associated with streams and water courses), high-lands (with rocky peaks which provide shadow during summer) and low production areas (which are eroded areas, with high proportions of bare ground and low levels of forage). Additionally, during those vegetation surveys we registered GPS locations of sheep carcasses and faeces from pumas and foxes to identify which vegetation units present higher predation risk (see Supplemental Information 1, Table S1.4).

For this study, we considered three paddocks, called *Repunte-Bajo (RB), Frison-Guanaco (FG)*and* Side (S)*, which have sizes of 79, 944 and 146 Ha, respectively. This range of sizes among paddocks is representative for the paddocks used commonly in Patagonian rangelands for sheep production (see examples in Irisarri et al., 2012; Ormaechea & Peri, 2015). Information about the surface of each landscape unit, the amount of forage species, bare ground and predation risk levels of each paddock can be found in Supplemental Information 1. Central and peripheral wetlands were considered the most preferred landscape resources because they contain higher forage production. Native forests have relatively high quantities of forage and riparian forests are areas associated with semi-permanent water courses, but are closed areas with lower probabilities of predator detection (Risenhoover & Bailey, 1985; Kie, 1999) and high numbers of carcasses and predators’ faeces (Supplemental Information 1, Table S1.4). Grasslands and shrubland-grasslands are areas with moderate to low quantities of forage and predation risk signs. High-lands are presented only at *Frison-Guanaco* and are characterized by protruding rock walls that sheep use as shaded areas during summer and as wind refuges during winter (A di Virgilio, pers. obs., 2014). Finally, low production areas are sites of moderate-low elevation with the lowest quantities of forage observed in the landscape.

#### Sheep data

We selected 19 sheep from a flock of approximately 200 individuals, composed of 38% of ewes (i.e., reproductive females), 28% of hoggets (i.e., female and male yearlings), 31% of wethers (i.e., castrated males), and 3% of rams (reproductive males). In order to have a representative sample of these sheep classes (rams were kept apart from the flock during the sampling period), we selected 6 ewes, 7 hoggets, and 6 wethers. Sheep were equipped with GPS devices attached to collars (CatLog-B, Perthold Engineering, www.perthold.de; USA). Collared animals were marked for identification with a unique ID number and colour according to sheep class. The sampled animals were always the same individuals for all the sampling periods, and sheep wore the collars almost continuously for the whole duration of the experiment. The devices were programmed to acquire locations every 5 min, from September 2014 to September 2015.We used a Body Condition Score (BCS, Jefferies, 1961; Giraudo, 2010), as an indirect method to assess sheep fat reserves and estimated sheep’ age by their number of permanent teeth. Initial BCS of sheep and age were measured at the beginning of the sampling period. BCS ranged from 2.25 to 4 and ages ranged from 6 months to more than 4 years old (i.e., from 0 permanent teeth to 8 permanent teeth). We also measured BSC of all collared individuals every time the flock was translocated into the paddocks. We considered heterogeneous flocks (hereafter 3-class mixed flocks) as those composed of all sheep classes (i.e., ewes, hoggets and wethers) and less heterogeneous flocks (hereafter 2-class mixed flocks) as flocks with two sheep classes (e.g., ewes and hoggets, or wethers and hoggets). Because our research was conducted on non-regulated animals we did not required any ethical approval.

To assess if patterns of space use by sheep are more evenly distributed when animals graze in 3-class mixed flocks and to detect which individuals have priority access to most preferred areas, we selected movement data from the periods when all sheep classes grazed together in the same paddock (see Table 1). During our sampling period, the flock was divided according to sheep classes (see Borrelli et al., 1997; Golluscio, Deregibus & Paruelo, 1998) during lambing (i.e., when ewes give birth to lambs, approximately between October–November) and during weaning (i.e., when wethers and lambs were separated, approximately between January–February). To evaluate if sheep increase their selectivity under less heterogeneous mixed flocks, and if subordinates change their landscape use patterns when high-ranked individuals are removed from the flock, we used the following data sets: (i) Data from wethers and hoggets grazing when ewes were extracted from the flock during the lambing period (RB_2C-mixed_, see Table 1); and (ii) data from ewes and hoggets grazing when wethers were extracted from the flock during weaning (S_2C-mixed_, see Table 1). Both databases included GPS locations of individuals that experienced both flock types in the same paddock.

**Table 1:** Description of datasets used for each analysis, including the paddock considered, the type of flock, the number of individuals included of each sheep class, and the period and its length in days. The data correspond to the same animals, and the difference in the total number of individuals occurs when some devices did not work because they had run out of batteries.

Database	Paddock	Flock type	Number of individuals	Period
RB_3C-mixed_	*Repunte Bajo*	3 classes: ewes, hoggets, and wethers	19 in total: 6 ewes, 7 hoggets and 6 wethers	September–November 2014 and April–May 2015 (Length: 77 days)
FG_3C-mixed_	*Frison-Guanaco*	3 classes: ewes, hoggets and wethers	18 in total: 6 ewes, 7 hoggets and 5 wethers	February–August 2015 (Length: 164 days)
*S*_3C-mixed_	*Side*	3 classes: ewes, hoggets and wethers	15 in total: 6 ewes, 5 hoggets and 4 wethers	September 2015 (Length: 30 days)
RB_2C-mixed_	*Repunte Bajo*	2 classes: wethers and hoggets	6 in total: 2 wethers and 4 hoggets	November–January 2014 (Length: 78 days)
*S*_2C-mixed_	*Side*	2 classes: ewes and hoggets	8 in total: 5 ewes and 3 hoggets	January and September 2015 (Length: 39 days)

In all cases, we used only diurnal locations when sheep are mainly grazing (Dudzinsky & Arnold, 1978). As a result, the total number of GPS locations to be included in the data analysis for *Repunte-Bajo*, *Frison-Guanaco* and *Side* paddocks were: 147,905, 208,275 and 28,976 respectively. The number of random points generated for statistical analyses (see below) was double of GPS locations for the three paddock, which represented a good balance between the quality of the availability estimation and computational demands (Northrup et al., 2013). The spatial data analysis was performed with R software (R Core Team, 2015). All information about spatial data analysis can be found in Supplemental Information 2.

### Data analysis

To assess sheep resource selection patterns at landscape scale, we focused our analyses on availability vs. use of resources (e.g., Johnson et al., 2006; Beyer et al., 2010), where different resources are represented by the different vegetation units present in the landscape. This requires measuring the use of resources relative to their availability: a positive ratio indicates that animals are selecting a particular resource, a negative ratio suggests avoidance, and the magnitude of the ratio indicates the degree of the selection/avoidance. In particular, we estimated Resource Selection Functions (RSF, Manly et al., 2002), by fitting logistic regressions, assuming a Bernoulli distribution for the response variable and a *logit* link function. We used a Bayesian approach to fit a hierarchical logistic regression for each paddock allowing for individual differences in sheep responses and considering the lack of independence between the consecutive GPS locations (see Supplemental Information 3). The dependent variable for the RSF was the GPS locations (ones) combined with simulated random locations (zeroes) across the total surface of each paddock that approximate the availability of resources. The resources considered were landscape vegetation units: low production areas, high-lands, central wetlands, peripheral wetlands, grasslands, shrubland-grasslands, native forestsand riparian forests.

To assess the effect of social structure on the use of landscape resources, we fitted a hierarchical logistic model using time since release (as the number of days since the release in the paddock), sheep class, age and body condition score as co-variables. Continuous variables were centred and standardized. The model structure can be found in Supplemental Information 3. To evaluate if sub-dominant individuals shift their space use patterns when dominant sheep are removed from the flock, we fitted a hierarchical logistic model using as independent variable time since release, sheep type and flock type (i.e., 3-class vs. 2-class); see the model structure in Supplemental Information 3. All analyses were performed using R (R Core Team, 2015) and WinBUGS (Spiegelhalter et al., 1996). Details on parameters’ and hyper-parameters’ priors and the number of iterations for each Monte Carlo Markov Chain (MCMC) can be found in Supplemental Information 3. We checked for convergence and autocorrelation of the posterior distributions and estimated the 95% Highest Posterior Density (HPD) intervals for all parameters. To assess if resource selection patterns differed among sheep classes, we calculated the proportion of posterior distribution overlapping of the parameters for each landscape resource between (i) ewes-hoggets, (ii) ewes-wethers, and (iii) hoggets-wethers in each paddock. To evaluate the differences of resource selection under both types of flocks, we calculated the proportion of posterior distribution overlapping between the estimates of 3-class and 2-class mixed flocks for each resource in each paddock.

## Results

**Figure 1:**
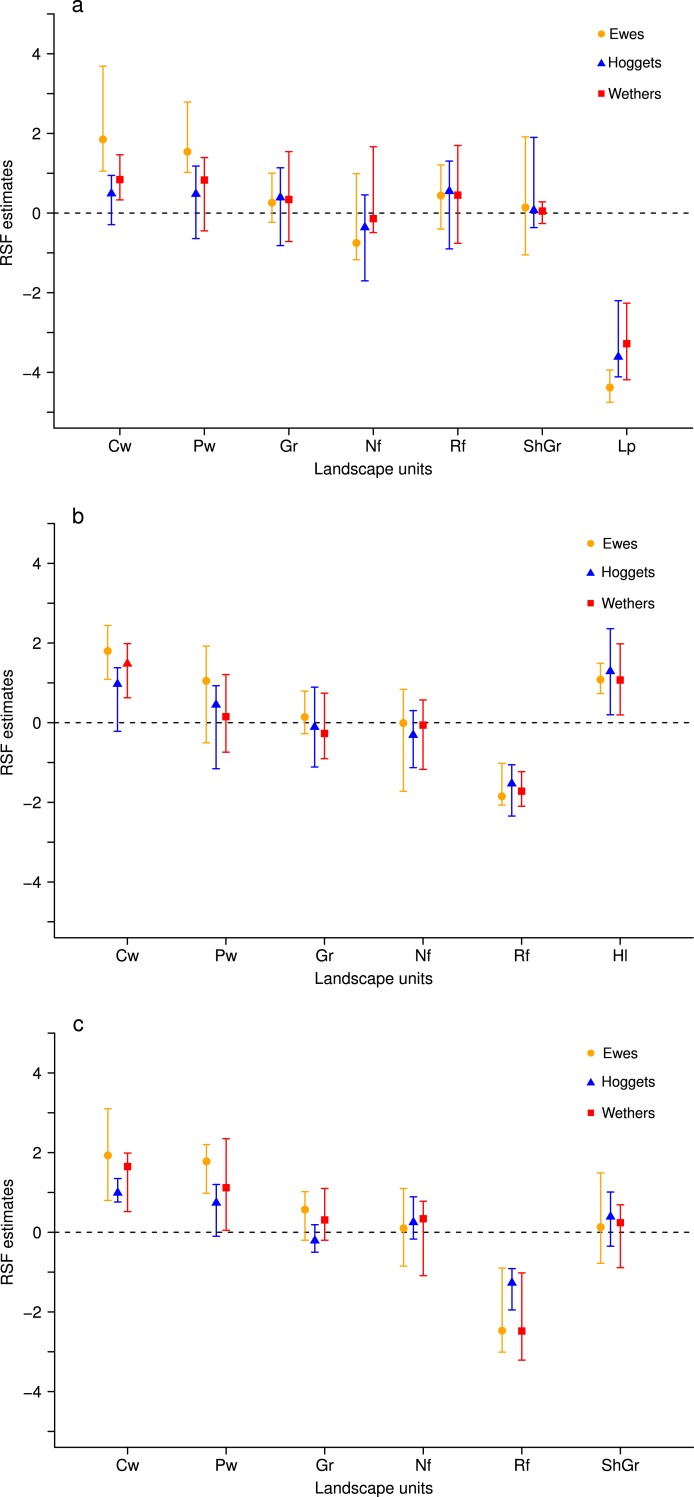
Resource Selection Functions mean estimates and 95% Highest Posterior Density intervals for the logistic model fitted to 3-class mixed flock for ewes (orange circles), hoggets (blue triangles) and wethers (red squares) grazing at the three paddocks. Estimated values located above zero (dashed line) indicate selection while values located below it indicate avoidance of the different vegetation units: low production (Lp), high-lands (Hl), central wetlands (Cw),peripheral wetlands (Pw), grassland (Gr), shrubland-grassland (ShGr), native forest (Nf) and riparian forest (Rf). At *Repunte-Bajo* (A) all sheep avoided low production areas, and the use of the rest of the vegetation units resulted similar, with the exception of central and peripheral wetlands that were the most selected habitats for ewes. At *Frison-Guanaco* (B) and *Side* (C) paddocks, all sheep avoided riparian forest and tended to use similarly all the habitats, with the exception of ewes that tended to select more central and peripheral wetlands in comparison to wethers and hoggets.

We found that when sheep were maintained in more heterogeneous flocks they selected almost all vegetation units with similar intensities and strongly avoided low production areas or riparian forests (Fig. 1). Furthermore, we found that for the areas with higher forage production (i.e., central and peripheral wetlands) and for less preferred areas (i.e., low production sites and riparian forests), the patterns of resource selection differed among sheep classes. This resulted in ewes selecting more intensively the most productive areas and avoiding with more strength the less preferred areas; while hoggets and wethers selected landscape resources similarly (Fig. 1, Supplemental Information 4: Table S4.1, Supplemental Information 5). We did not detect effects of time since release, age or BCS on sheep selection patterns (RSF coefficients were close to zero and HPD-intervals included zero; see Supplemental Information 4, Table S4.2). These patterns of more evenly distributed use and differences among sheep classes were observed at the three paddocks evaluated (Figs. 1 and 4, Supplemental Information 5).

**Figure 2:**
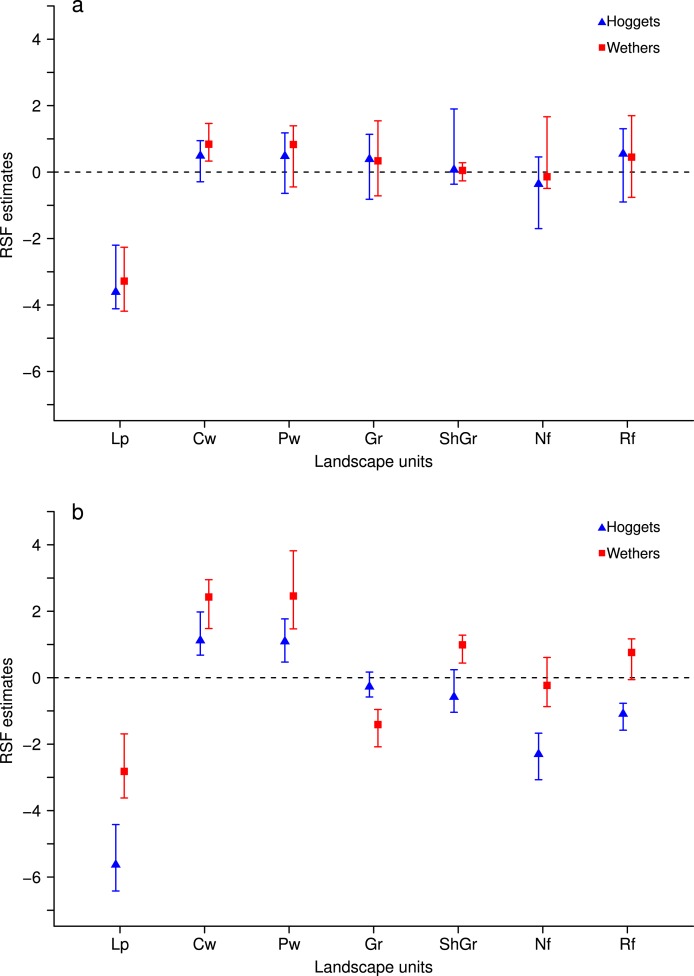
Resource Selection Function mean estimates and 95% Highest Posterior Density intervals for the logistic model fitted to data from hoggets (blue triangles) and wethers (red squares) grazing in 3-class (A) and 2-class (B) mixed flocks after ewes were removed at *Repunte-Bajo* paddock. Estimated values located above zero indicate selection and values located below it indicate avoidance of the different vegetation units: low production (Lp), central wetlands (Cw), peripheral wetlands (Pw), grassland (Gr), shrubland-grassland (ShGr), native forest (Nf) and riparian forest (Rf). We can see that, when extracting ewes from the flock (B), wethers and hoggets selected with more intensity central and peripheral wetlands; and hoggets strongly avoided low production areas, native and riparian forest.

**Figure 3:**
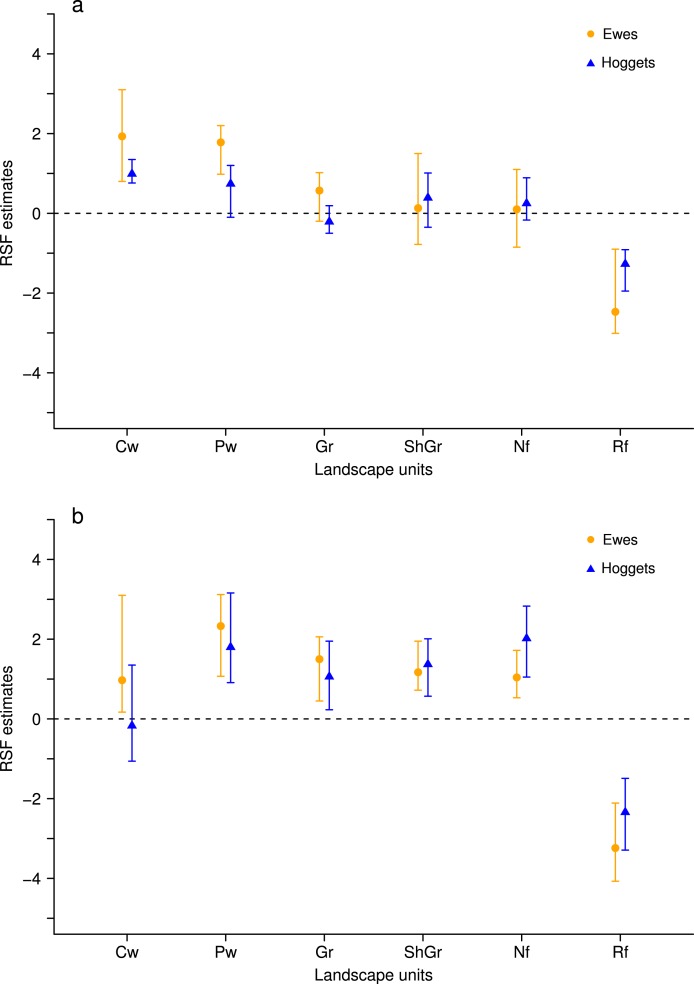
Resource Selection Function mean estimates and 95% Highest Posterior Density intervals for the logistic model fitted to data from hoggets (blue triangles) and ewes (orange circles) when grazing in a 3-class mixed flock (A) and in a 2-class mixed flock after wethers were removed (B) at *Side* paddock. Estimated values located above zero indicate selection and values located below it indicate avoidance of the different vegetation units: central wetlands (Cw), peripheral wetlands (Pw), grassland (Gr), shrubland-grassland (ShGr), native forest (Nf) and riparian forest (Rf). We can observe that when wethers were extracted from the flock (B) the general pattern of resource reselection did not change considerably: Ewes continued selecting with more intensity central and peripheral wetlands and that both sheep classes avoided riparian forest, and used the rest of the landscape units similarly.

**Figure 4:**
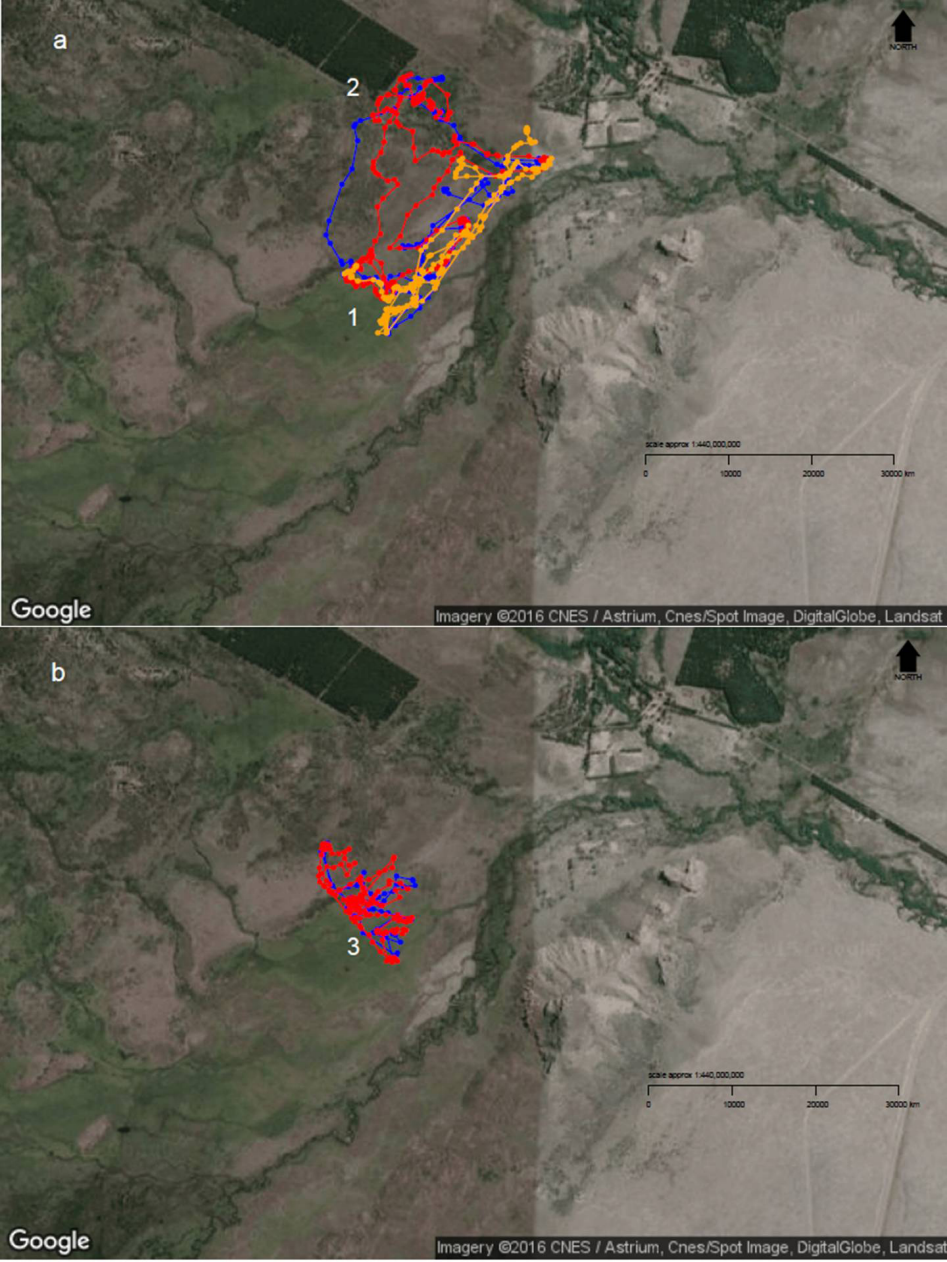
Diurnal movement patterns of sheep under 3-category and 2-category mixed flocks at *Repunte-Bajo* paddock. When sheep were grazing in a 3-category mixed flock (A), we can see that, although the ewe (orange path) showed a directed path towards the most preferred area (1), the other two classes showed a more distributed space use (2). After ewes were removed from the landscape (B), wethers (red path) and hoggets (blue path), concentrated their movements on the central and peripheral wetlands (3), which are the most preferred areas. To view more images of sheep movement patterns, see Supplemental Information 5. Map Data: Google, CNES/Astrium, Cnes/Spot Image, DigitalGlobe, Landsat.

When sheep were kept in less heterogeneous flocks, we noticed that the outcome of the resource selection patterns depended on which sheep class was removed (Figs. 2 and 3). When wethers were removed, the selection patterns resulted similar to those in 3-class mixed flocks (Figs. 3A and 3B), and neither ewes nor hoggets showed major changes in their landscape use. Nevertheless, when ewes were removed from the flock, it resulted in a more unevenly distributed space use, with higher selection of most preferred areas (i.e., central and peripheral wetlands) and higher avoidance of the rest of the landscape units (especially for low production areas and both types of forests). Moreover, hoggets were the sheep class that showed a marked switch in their resource selection patterns in comparison to wethers (Figs. 2A, 2B and 4; Supplemental Information 5).

## Discussion

This work provides information about a possible mechanism underlying the generation of more evenly distributed grazing patterns, which is related to ecological and behavioural aspects of livestock and can be employed as a tool for the development of sustainable livestock management strategies. Our outcomes showed that maintaining flocks of sheep from different classes resulted in a more homogeneous distribution of grazing, independently of the paddock in which sheep grazed. On the other hand, we observed that this evenly distributed grazing patterns could be easily overridden by common management practices that tend to maintain more uniform groups to increase flock productivity (Arnold & Pahl, 1974; Stenseth, 1983; Lynch et al., 1989; Borrelli et al., 1997; Borrelli & Oliva, 2001), such as flock segregation according to nutritional requirements or culling, among others. Finally, we noticed a shift from an evenly distributed grazing pattern toward a more selective one only when dominant individuals were removed from the flock.

Although there is evidence that food preferences, weather, forage distribution and predators influence herbivores grazing patterns (Scott, Provenza & Banner, 1995; Prache, Gordon & Rook, 1998), we propose that social interactions are also important factors influencing landscape use and ultimately population dynamics (Morales et al., 2010). This is particularly relevant for gregarious animals like livestock and we believe that it should be considered into management strategies in addition to the rest of the factors. Commonly, social interactions among group members result in dominance structures or social ranks (Lynch, Hinch & Adams, 1992; Erhard et al., 2004) that are reflected in their spatial distribution when grazing (see Lynch, Hinch & Adams, 1992; Sibbald, Shellard & Smart, 2000). We observed that ewes selected with more intensity the most preferred areas in comparison to hoggets and wethers that showed higher selectivity towards less preferred areas when sheep were maintained in more heterogeneous flocks. This pattern could be indicating the development of a well established social rank, in which ewes are dominant and have preferential access to resources, as was previously observed in other studies with sheep (Lynch, Wood-Gush & Davies, 1985; Eccles & Shackleton, 1986; Festa-Bianchet, 1991; Færevik, Andersen & Bøe, 2005), and hoggets and wethers occupied lower positions in the social rank.

Generally, to avoid aggressive interactions, subordinate individuals use the resources after dominants and are forced to feed on less preferred areas (Eccles & Shackleton, 1986; Prache, Gordon & Rook, 1998) while high-ranked animals are generally unaffected by the proximity of subordinates (e.g., Bennett, Finch & Holmes, 1985). This would mean that low-ranked individuals are more limited on their choices when high-ranked individuals are present. Under this context, we would expect that, when these dominant individuals are removed from the flock, lower ranked individuals respond by showing a switch in their selection patterns toward the most preferred areas. Although some studies focused on dominance levels of wethers (Stolba et al., 1990; Keeling, 2001) there is not a conclusive statement about their position in social ranks. In this work, we did not observed any switch in the landscape use patterns of hoggets and ewes when wethers were extracted from the flock. Moreover, when ewes were removed, only hoggets showed a significant shift in their selectivity, strongly selecting most preferred areas and avoiding less preferred areas. These results imply that reproductive females are upper positioned in the social rank, maybe due to their higher energetic demands (Keeling, 2001); wethers are more or less independent of this social rank maybe associated to their relatively constant energetic demands generated by castration, and immature individuals are at the lowest levels of this ranking.

Although there is evidence that age and body condition can be factors influencing animals’ resource selection and their social interactions (Festa-Bianchet, 1991; Côté, 2000; Veiberg et al., 2004; Vervaecke, Roden & De Vries, 2005), we did not find evidence about the potential effects of these factors on the landscape use of our sheep. This lack of evidence may be due to the fact that we did not have enough variation in age or body condition score among sampled individuals, or because we would need a bigger sample to assess those effects. Studies that found an effect of age and body condition on sheep social rank were conducted on homogeneous flocks composed mostly of one sheep class and under pen or small paddock conditions instead of rangelands. Nevertheless, the lack of effect of body condition could have important implications on livestock productivity, because if sub-dominant individuals would be severely food restricted under more heterogeneous flocks, we would expect a strong association between body condition score and the use of less preferred areas and it was not the case in our results.

Unevenly distributed grazing, besides generating degradation of over-used areas, decreases the productivity of under-grazed parts of the landscape mainly because of the accumulation of senescent material (Belsky, 1986). Thus, if grazing distribution could be controlled, we could consider it as a potential tool for natural grasslands conservation and recovery (see Weber et al., 1998; Adler, Raff & Lauenroth, 2001). Even though the stocking rates were reduced when sheep shifted from a 3-class to a 2-class mixed flock, if our findings would have been related to the number of animals in a flock, we would expect to find similar results if we would have removed ewes as we would have removed wethers, because all sheep classes represented similar proportions of the flock. Nevertheless, we observed a marked switch in grazing patterns, only after ewes were removed. For this reason, we propose that social interactions among flock members is the underlying mechanism that generates more evenly distributed grazing patterns and the disruption of this social rank generates highly selective grazing patterns. Considering that livestock resource selection patterns affect not only animals’ nutritional levels (Stricklin, Zhou & Gonyou, 1995) but also landscape dynamics (Adler, Raff & Lauenroth, 2001), our findings have important management implications and provide information that could be used to decrease the negative impacts of sheep grazing and maintain the long-term productivity of natural grasslands.

## Conclusions

Several management tools have been proposed to homogenize livestock grazing patterns, such as water developments (Porath et al., 2002), fencing (Bailey, Walker & Rittenhouse, 1990), strategic supplement placement (Bailey et al., 2001), herding (Rhodes & Marlow, 1997), and stocking density increasing (Savory, 1983). However, most of these tools require relatively high investments (e.g., Bailey, 2004) or resulted controversial (Briske et al., 2013; Cibils et al., 2014; Teague, 2014). We are aware that the adoption of a novel management strategy strongly depends on ecological and economical constraints, and that social and cultural factors play a key role in this process (Borrelli et al., 1997; Golluscio, Deregibus & Paruelo, 1998). In this work, we are suggesting to land managers and livestock producers an alternative for flock management that employs animals’ behavioural interactions without the need for reducing livestock rates, modifying stocking densities or additional investments in labour and materials. Moreover, managing more heterogeneous mixed flocks can be combined with other strategies such as rotational grazing and the use of guardian dogs (to decrease the mortality levels that may be generated by sheep grazing on more risky habitats) to increase livestock productivity and landscape conservation.

##  Supplemental Information

10.7717/peerj.2152/supp-1 Supplemental Information 1 Landscape description

10.7717/peerj.2152/supp-2 Supplemental Information 2 Spatial data processing

10.7717/peerj.2152/supp-3 Supplemental Information 3 Model structure

10.7717/peerj.2152/supp-4 Supplemental Information 4 Model output

10.7717/peerj.2152/supp-5 Supplemental Information 5 Movement paths and GPS locations

## Additional Information and Declarations

### Field Study Permissions

The following information was supplied relating to field study approvals (i.e., approving body and any reference numbers): The vegetation data used in this work is shared with another project and researchers, for which we have applied for permission to National Parks Administration (Permit number 1399). The permit (in Spanish) and an English summary is attached in this submission.

### Data Availability

The following information was supplied regarding data availability: The data can be freely downloaded by following these steps: (1) Clicking on the projects map (https://www.movebank.org/panel_embedded_movebank_webapp) and searching the project (by key words such as Ovis aries Patagonia or by the entire project name: Ovis aries di Virgilio Patagonia); (2) selecting the study (by clicking on the green point representing our study); (3) opening the study page (by clicking on “Open in study page”); and (4) clicking on “Download” tab. After reading the study license terms, the data can be freely downloaded in different file formats.
