# Supplementary material for: Towards evenly distributed grazing patterns: including social context in sheep management strategies
Source: PeerJ. 2016 Jun 21;4:e2152. doi: 10.7717/peerj.2152 (PMC4924134; doi:10.7717/peerj.2152)
Supplement: Supplemental Information 1 [file peerj-04-2152-s001.docx]

Appendix S1: Landscape description.

Figure 1: Study area location in Argentina (a). Location of Fortin Chacabuco Ranch (b, c):The study site is located in a transition area between Sub-Andean temperate forests and Patagonian steppes inside the National Park Nahuel Huapi. The full extension of the farm is approximately 4300 ha and consisted in several paddocks of different sizes ranging from 4 ha to up to 1000 ha. The three paddocks used in this study were *Repunte Bajo*, *Frison-Guanaco* and *Side* (d).


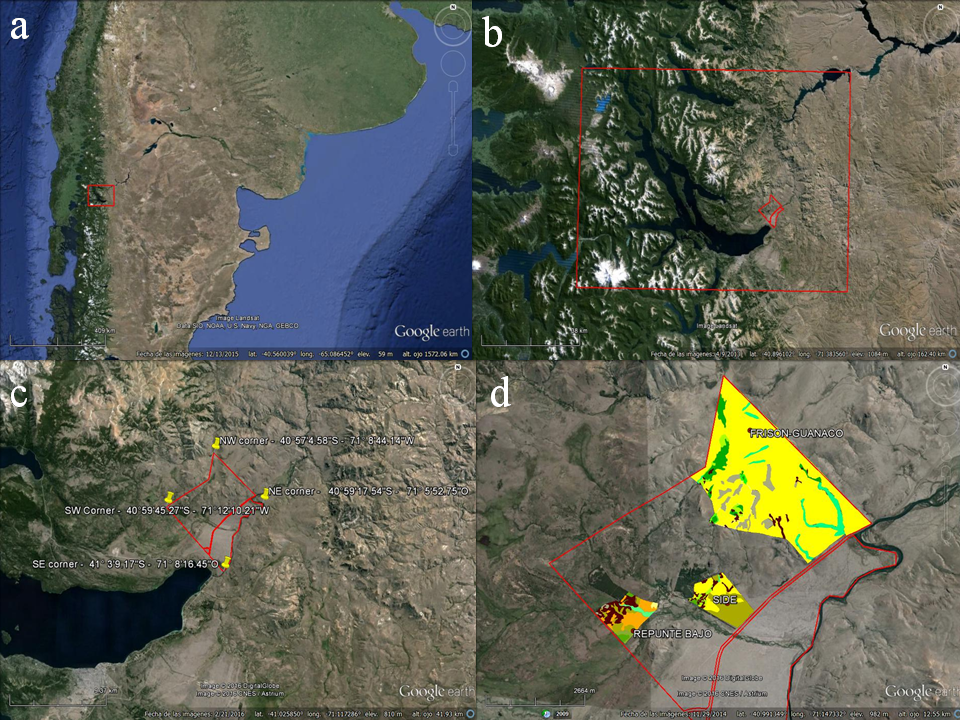


Table S1.1: Total area of each paddock in hectares and percent cover of each landscape unit considered the analyses: low production areas (Lp), high-lands (Hl), central wetlands (Cw), peripheral wetlands (Pw), grasslands (Gr), shrubland-grasslands (ShGr), native forests (Nf) and riparian forests (Rf)

| Paddock | Total area (Ha.) | Landscape unit (%) | | | | | | | |
| --- | --- | --- | --- | --- | --- | --- | --- | --- | --- |
|  |  | Lp | Hl | Cw | Pw | Gr | ShGr | Nf | Rf |
| *Repunte Bajo* | 79.00 | 0.16 | 0.00 | 0.05 | 0.10 | 0.20 | 0.16 | 0.30 | 0.01 |
| *Frison-Guanaco* | 944.00 | 0.00 | 0.37 | 0.01 | 0.06 | 0.34 | 0.00 | 0.19 | 0.03 |
| *Side* | 146.00 | 0.00 | 0.00 | 0.03 | 0.02 | 0.61 | 0.28 | 0.05 | 0.01 |

Table S1.2: Percent relative cover of palatable species in each landscape unit and each paddock: low production areas (Lp), high-lands (Hl), central wetlands (Cw), peripheral wetlands (Pw), grasslands (Gr), shrubland-grasslands (ShGr), native forests (Nf) and riparian forests (Rf). NA values indicate Non-Available data for a particular landscape unit, because it is absent in the paddock

| Paddock | Landscape unit | | | | | | | | | | | | | | | |  |
| --- | --- | --- | --- | --- | --- | --- | --- | --- | --- | --- | --- | --- | --- | --- | --- | --- | --- |
|  | Lp | | Hl | | Cw | | Pw | | Gr | | ShGr | | Nf | | Rf | |  |
| *Repunte Bajo* | | 1.50 | | NA | | 34.87 | | 19.95 | | 11.16 | | 7.36 | | 21.43 | | 19.57 | |
| *Frison-Guanaco* | | NA | | 8.00 | | 35.84 | | 16.70 | | 11.95 | | NA | | 15.32 | | 9.96 | |
| *Side* | | NA | | NA | | 23.19 | | 17.13 | | 6.99 | | 5.00 | | 20.95 | | 36.71 | |

Table S1.3: Percentage of bare ground in each landscape unit of each paddock: low production areas (Lp), high-lands (Hl), central wetlands (Cw), peripheral wetlands (Pw), grasslands (Gr), shrubland-grasslands (ShGr), native forests (Nf) and riparian forests (Rf). NA values indicate Non-Available data for a particular landscape unit, because it is absent in the paddock

| Paddock | Landscape unit (Ha) | | | | | | | | | | | | | | | |  |
| --- | --- | --- | --- | --- | --- | --- | --- | --- | --- | --- | --- | --- | --- | --- | --- | --- | --- |
|  | Lp | | Hl | | Cw | | Pw | | Gr | | ShGr | | Nf | | Rf | |  |
| *Repunte Bajo* | | 97.78 | | NA | | 1.00 | | 3.00 | | 30.67 | | 9.67 | | 12.41 | | 0.00 | |
| *Frison-Guanaco* | | NA | | 49.17 | | 2.50 | | 18.50 | | 66.08 | | NA | | 1.00 | | 0.00 | |
| *Side* | | NA | | NA | | 0.00 | | 2.00 | | 14.71 | | 15.00 | | 1.50 | | 1.00 | |

Table S1.4: Proportions of risky locations (i.e., GPS locations of sheep carcasses and feces from puma and foxes) in each landscape unit of each paddock: low production areas (Lp), high-lands (Hl), central wetlands (Cw), peripheral wetlands (Pw), grasslands (Gr), shrubland-grasslands (ShGr), native forests (Nf) and riparian forests (Rf). NA values indicate Non-Available data for a particular landscape unit, because it is absent in the paddock

| Paddock | Landscape unit | | | | | | | | | | | | | | | |  |
| --- | --- | --- | --- | --- | --- | --- | --- | --- | --- | --- | --- | --- | --- | --- | --- | --- | --- |
|  | Lp | | Hl | | Cw | | Pw | | Gr | | ShGr | | Nf | | Rf | |  |
| *Repunte Bajo* | | 0.00 | | NA | | 0.00 | | 0.00 | | 0.07 | | 0.21 | | 0.43 | | 0.29 | |
| *Frison-Guanaco* | | NA | | 0.00 | | 0.00 | | 0.00 | | 0.17 | | NA | | 0.33 | | 0.5 | |
| *Side* | | NA | | NA | | 0.00 | | 0.64 | | 0.09 | | 0.00 | | 0.18 | | 0.09 | |
