# Supplementary material for: Towards evenly distributed grazing patterns: including social context in sheep management strategies
Source: PeerJ. 2016 Jun 21;4:e2152. doi: 10.7717/peerj.2152 (PMC4924134; doi:10.7717/peerj.2152)
Supplement: Supplemental Information 2 [file peerj-04-2152-s002.docx]

Appendix S2: Steps of Spatial Data Processing:

1. The first step consisted in filtering GPS data to exclude nocturnal locations.We developed a function that considered the time (in hours, minutes and seconds) of sunset and nightfall according to the date and geographic location, to select only the GPS locations between these two times.

2. Once we have our diurnal data set, we used the R functions *SpatialPoints* and *SpatialPointsDataFrame* from sp package (Pebesma & Bivand 2005), to transform GPS coordinates in a spatial object with attributes of latitude, longitude and point index. In this step of processing we defined the coordinate reference system for our data: datum WGS84, and projection UTM 19 South.

3. We used the R function *readShapePoly* from maptools package (Bivand & Lewin-Koh 2015) and *SpatialPolygonsDataFrame* from sp package, to read the polygon shape filesand use the landscape unit information from each paddock. The coordinate reference system for polygons was also WGS84, UTM 19 South.

4. The fourth step consisted in generating random points distributed across the paddocks to estimate landscape resource availability (see Data Analysis in the main text) using the R function *spsample* from sp package.

5. We converted these random locations into a sp object, repeating the same procedure than in step 2.

6. We overlapped each point (GPS locations and random points) with their correspondent polygon shapefile using the function *over* from sp package. This last step allowed us to extract for each point the corresponding type of landscape unit.

References

Pebesma, E.J., & Bivand, R.S. (2005) Classes and methods for spatial data in R. R News 5 (2), <http://cran.r-project.org/doc/Rnews/>.

Bivand, R. & Lewin-Koh, N. (2015) maptools: Tools for Reading and Handling Spatial Objects. R package version 0.8-37. <http://CRAN.R-project.org/package=maptools>
