# Supplementary material for: Towards evenly distributed grazing patterns: including social context in sheep management strategies
Source: PeerJ. 2016 Jun 21;4:e2152. doi: 10.7717/peerj.2152 (PMC4924134; doi:10.7717/peerj.2152)
Supplement: Supplemental Information 3 [file peerj-04-2152-s003.docx]

Appendix S3: Model Structure

Equation S3.1: The following equation represents the model structure to characterize the use of the different vegetation units present in each paddock according to *(1)* sheep category, *(2)* body condition score, *(3)* age of the individuals, and *(4)* time (as the number of days since the release in the paddock). The response variable *location* is composed by GPS and random points (1 and 0 values, respectively). The explanatory variable *resource* represents each landscape unit (i.e., Lp: Low production; Hl: High-land; Cw: Central wetland; Pw: Peripheral wetland; Gr: Grassland; ShGr: Shrubland-Grassland; Nf: Native forest; Rf: Riparian forest). Parameter *beta_0_* represents the selection of the reference landscape unit, which was low production area in *Repuntebajo* paddock, High-lands in *Frison-Guanaco* paddock and central wetland in *Side* paddock. The parameters *beta_k_* represent the rest of the landscape units present in each paddock (with *k* ranging from 1 to the total number of landscape units present in each paddock K). Parameters α*_0_*and α*_k_* represent the selection of ewes; β*_0_* and β*_k_* represent the selection of hoggets; γ*_0_* and γ*_k_* represent the selection of wethers. The sign of these parameters indicates if sheep are selecting (positive sign) or avoiding (negative sign) a particular resource and the value indicate the strength of that selection/avoidance. Parameter $\psi$ represents the effect of time since release, $\delta$ the effect of age and $\lambda$ the effect of body condition score on the resource selection patterns. In the model, subscript *i* represents each observation and *j* represents each individual. We fitted a separated model for each paddock, run 20.000 iterations for three chains and used a burn-in of 10.000 iterations.

$$location_{ij}\sim Bernoulli \left( p_{ij} \right)$$

$$logit\left( p_{ij} \right)= {beta}_{0j}+ \sum_{k=1}^{K} {beta}_{kj}\times R_{i}+ \psi_{j} \times T_{i}$$

$${beta}_{0j}\sim Normal \left( \mu_{0j},\sigma_{0} \right)$$

$${beta}_{kj}\sim Normal \left( \mu_{kj},\sigma_{k} \right)$$

$$\psi_{j}\sim Normal \left( \mu_{\psi j},\sigma_{\psi} \right)$$

$$\mu_{beta0,j}= \alpha_{0}+ {}_{0}*{hoggets}_{j}+ {}_{0}*{wethers}_{j}+ \delta_{0}*{age}_{j}{+ \lambda}_{0}*{cc}_{j}$$

$$\mu_{betak,j}= \alpha_{k}+ {}_{k}*{hoggets}_{j}+ {}_{k}*{wethers}_{j}+ \delta_{k}*{age}_{j}{+ \lambda}_{k}*{cc}_{j}$$

We have chosen vague prior distribution for all parameters ad hyper-parameters in the model:

$$\alpha_{0} \sim Normal (0, 2)$$

$$\alpha_{k} \sim Normal (0, 2)$$

$${}_{k} \sim Normal (0, 2)$$

$${}_{k} \sim Normal (0, 2)$$

$$\delta_{k} \sim Normal (0, 2)$$

$$\lambda_{k}\sim Normal (0, 2)$$

$$\mu_{\psi}\sim Normal (0, 2)$$

$$\sigma_{0}\sim Uniform (0, 10)$$

$$\sigma_{k}\sim Uniform \left( 0, 10 \right)$$

$$\sigma_{\psi}\sim Uniform (0, 10)$$

Equation S3.2: The following equation represents the model structure to characterize the use of the different vegetation units present in each paddock according to *(1)* sheep category, *(2)* body condition score, *(3)* age of the individuals, and *(4)* time (as the number of days since the release in the paddock). The response variable *location* is composed by GPS and random points (1 and 0 values, respectively). The explanatory variable *resource* represents each landscape unit (i.e., Lp: Low production; Hl: High-land; Cw: Central wetland; Pw: Peripheral wetland; Gr: Grassland; ShGr: Shrubland-Grassland; Nf: Native forest; Rf: Riparian forest). Parameter *beta_0_* represents the selection of the reference landscape unit, which was low production area in *Repunte bajo* paddock, High-lands in *Frison-Guanaco* paddock and central wetland in *Side* paddock. The parameters *beta_k_* represent the rest of the landscape units present in each paddock (with *k* ranging from 1 to the total number of landscape units present in each paddock K). Parameters α*_0_*and α*_k_* represent the selection of ewes; β*_0_* and β*_k_* represent the selection of hoggets; γ*_0_* and γ*_k_* represent the selection of wethers. The sign of these parameters indicates if sheep are selecting (positive sign) or avoiding (negative sign) a particular resource and the value indicate the strength of that selection/avoidance. Parameter $\psi$ represents the effect of time since release, and ${}_{k}$ the effect of 2-category mixed flocks. Parameters α*_0_* and α*_k_* represent the selection of ewes; β*_0_* and β*_k_* represent the selection of hoggets; γ*_0_* and γ*_k_* represent the selection of wethers. The sign of these parameters indicates if sheep are selecting (positive sign) or avoiding (negative sign) a particular resource and the value indicate the strength of that selection/avoidance. In the model, subscript *i* represents each observation and *j* represents each individual. We fitted a separated model for each paddock, run 20.000 iterations for three chains and used a burn-in of 10.000 iterations.

$$location_{ij}\sim Bernoulli \left( p_{ij} \right)$$

$$logit\left( p_{ij} \right)= {beta}_{0j}+ \sum_{k=1}^{K} {beta}_{kj}\times R_{i}+ \psi_{j} \times T_{i}$$

$${beta}_{0j}\sim Normal \left( \mu_{0j},\sigma_{0} \right)$$

$${beta}_{kj}\sim Normal \left( \mu_{kj},\sigma_{k} \right)$$

$$\psi_{j}\sim Normal \left( \mu_{\psi j},\sigma_{\psi} \right)$$

$$\mu_{beta0,j}= \alpha_{0}+ {}_{0}*{hoggets}_{j}+ {}_{0}*{wethers}_{j}+ {}_{k}*F_{j}$$

$$\mu_{betak,j}= \alpha_{k}+ {}_{k}*{hoggets}_{j}+ {}_{k}*{wethers}_{j}+ {}_{k}*F_{j}$$

We have chosen vague prior distribution for all parameters ad hyper-parameters in the model:

$$\alpha_{0} \sim Normal (0, 2)$$

$$\alpha_{k} \sim Normal (0, 2)$$

$${}_{k} \sim Normal (0, 2)$$

$${}_{k} \sim Normal (0, 2)$$

$${}_{k} \sim Normal (0, 2)$$

$$\mu_{\psi}\sim Normal (0, 2)$$

$$\sigma_{beta0}\sim Uniform (0, 10)$$

$$\sigma_{betak} \sim Uniform (0, 10)$$

$$\sigma_{\psi}\sim Uniform (0, 10)$$
