# Supplementary material for: Towards evenly distributed grazing patterns: including social context in sheep management strategies
Source: PeerJ. 2016 Jun 21;4:e2152. doi: 10.7717/peerj.2152 (PMC4924134; doi:10.7717/peerj.2152)
Supplement: Supplemental Information 4 [file peerj-04-2152-s004.docx]

Appendix S4: Model output

Table S4.1: Proportion of overlap between the posterior distributions of the habitat selection coefficients of ewes, hoggets and wethers at each paddock. The resources/habitats considered were: low production areas (Lp), high-lands (Hl), central wetlands (Cw), peripheral wetlands (Pw), grasslands (Gr), shrubland-grasslands (ShGr), native forests (Nf) and riparian forests (Rf). A high proportion of overlapping between the estimated posteriors indicate that sheep are selecting resources in a similar way and lower values of overlap indicates more differences in the resource selection. Values in bold and italic indicate overlaps equal or smaller than 0.25 of the posterior distribution

| Paddock | Landscape  unit | Overlap: Ewe/Hogget | Overlap: Ewe/Wether | Overlap: Hogget/Wether |
| --- | --- | --- | --- | --- |
| *Repunte Bajo* | Lp | ***0.00*** | ***0.00*** | 0.99 |
| *Repunte Bajo* | Cw | ***0.00*** | ***0.00*** | 0.96 |
| *Repunte Bajo* | Pw | ***0.01*** | ***0.00*** | 0.99 |
| *Repunte Bajo* | Gr | ***0.21*** | ***0.21*** | 0.97 |
| *Repunte Bajo* | ShGr | 0.82 | 0.86 | 0.92 |
| *Repunte Bajo* | Nf | ***0.22*** | 0.33 | 0.99 |
| *Repunte Bajo* | Rf | 0.33 | 0.51 | 0.91 |
| *Frison-Guanaco* | Hl | ***0.20*** | ***0.04*** | 0.96 |
| *Frison-Guanaco* | Cw | 0.40 | ***0.22*** | 0.99 |
| *Frison-Guanaco* | Pw | ***0.25*** | 0.49 | 0.95 |
| *Frison-Guanaco* | Gr | 0.98 | 0.49 | 0.98 |
| *Frison-Guanaco* | Nf | 0.934 | 0.99 | 0.99 |
| *Frison-Guanaco* | Rf | 0.31 | 0.39 | 0.99 |
| *Side* | Cw | ***0.09*** | ***0.25*** | 0.36 |
| *Side* | Pw | 0.93 | 0.95 | 0.99 |
| *Side* | Gr | 0.92 | 0.98 | 0.92 |
| *Side* | ShGr | 0.99 | 0.98 | 0.98 |
| *Side* | Nf | ***0.13*** | 0.98 | ***0.09*** |
| *Side* | Rf | 0.41 | ***0.18*** | 0.99 |

Table S4.2: Estimates for the effects of Age (δ*_k_*) Body Condition Score (BCS, λ*_k_*) and Time (ψ) on the resource selection patterns for sheep in *Repunte Bajo* paddock. The values below correspond to the mean and standard deviation (Sd) of the posterior distribution for each parameter, and the lower and upper values of the 95% HPD intervals. The landscape units considered as resources were low production areas (Lp), central wetlands (Cw), peripheral wetlands (Pw), grasslands (Gr), shrubland-grasslands (ShGr), native forests (Nf) and riparian forests (Rf)

| Repunte bajo estimates | | | | |
| --- | --- | --- | --- | --- |
| Parameter | Mean | Sd | 95% HPD interval | |
|  |  |  | Lower | Upper |
| δ_0_: Lp Age | -0.35 | 0.30 | -0.99 | 0.18 |
| δ_1_: Cw Age | 0.19 | 0.33 | -0.42 | 0.91 |
| δ_2_: Pw Age | 0.31 | 0.32 | -0.29 | 0.94 |
| δ_3_: Gr Age | 0.34 | 0.29 | -0.18 | 0.98 |
| δ_4_: ShGr Age | 0.35 | 0.31 | -0.19 | 0.99 |
| δ_5_: Nf Age | 0.44 | 0.32 | -0.14 | 1.11 |
| δ_6_: Rf Age | 0.39 | 0.30 | -0.14 | 1.03 |
| λ_0_: Lp BCS | -0.03 | 0.23 | -0.46 | 0.42 |
| λ_1_: Cw BCS | 0.08 | 0.24 | -0.39 | 0.55 |
| λ_2_: Pw BCS | 0.00 | 0.236 | -0.42 | 0.45 |
| λ_3_: Gr BCS | 0.04 | 0.226 | -0.39 | 0.45 |
| λ_4_: ShGr BCS | 0.01 | 0.24 | -0.45 | 0.43 |
| λ_5_: NF BCS | -0.03 | 0.24 | -0.48 | 0.43 |
| λ_6_: Rf BCS | -0.01 | 0.23 | -0.47 | 0.43 |
| ψ: Time | -0.01 | 0.01 | -0.02 | -0.01 |

Table S4.3: Estimates for the effects of Age (δ*_k_*) Body Condition Score (BCS, λ*_k_*) and Time (ψ) on the resource selection patterns for sheep in *Frison-Guanaco* paddock. The values below report the mean and standard deviation (Sd) of the posterior distribution for each parameter, and the lower and upper values of the 95% HPD intervals. The landscape units considered as resources were high-lands (Hl), central wetlands (Cw), peripheral wetlands (Pw), grasslands (Gr), native forests (Nf) and riparian forests (Rf). Values in bold and italic indicate HPD intervals that do not include zero

| *Frison-Guanaco* estimates | | | | |
| --- | --- | --- | --- | --- |
| Parameter | Mean | Sd | 95% HPD interval | |
|  |  |  | Lower | Upper |
| δ_0_: Hl Age | 0.08 | 0.15 | -0.24 | 0.38 |
| δ_1_: Cw Age | -0.12 | 0.22 | -0.56 | 0.28 |
| δ_2_: Pw Age | 0.00 | 0.14 | -0.26 | 0.28 |
| δ_3_: Gr Age | 0.05 | 0.09 | -0.13 | 0.24 |
| δ_5_: Nf Age | -0.09 | 0.28 | -0.64 | 0.43 |
| δ_6_: Rf Age | 0.08 | 0.23 | -0.36 | 0.54 |
| λ_0_: Hl BCS | -0.08 | 0.11 | -0.31 | 0.14 |
| λ_1_: Cw BCS | 0.15 | 0.15 | -0.17 | 0.47 |
| λ_2_: Pw BCS | 0.11 | 0.09 | -0.07 | 0.29 |
| λ_3_: Gr BCS | 0.05 | 0.07 | -0.08 | 0.20 |
| λ_5_: NF BCS | 0.16 | 0.23 | -0.28 | 0.59 |
| λ_6_: Rf BCS | 0.00 | 0.01 | -0.35 | 0.32 |
| ψ: Time | 0.01 | 0.01 | ***0.01*** | ***0.01*** |

Table S4.4: Estimates for the effects of Age (δ*_k_*) Body Condition Score (BCS, λ*_k_*) and Time (ψ) on the resource selection patterns for sheep in *Side* paddock. The values below report the mean and standard deviation (Sd) of the posterior distribution for each parameter, and the lower and upper values of the 95% HPD intervals. The landscape units considered as resources were central wetlands (Cw), peripheral wetlands (Pw), grasslands (Gr), shrubland-grasslands (ShGr), native forests (Nf) and riparian forests (Rf)

| *Side* estimates | | | | |
| --- | --- | --- | --- | --- |
| Parameter | Mean | Sd | 95% HPD interval | |
|  |  |  | Lower | Upper |
| δ_0_: Cw Age | -0.08 | 0.16 | -0.41 | 0.23 |
| δ_2_: Pw Age | 0.23 | 0.28 | -0.38 | 0.78 |
| δ_3_: Gr Age | -0.14 | 0.36 | -0.88 | 0.59 |
| δ_4_: ShGr Age | 0.06 | 0.25 | -0.45 | 0.55 |
| δ_5_: Nf Age | 0.06 | 0.14 | -0.22 | 0.35 |
| δ_6_: Rf Age | -0.17 | 0.55 | -1.25 | 0.94 |
| λ_0_: Cw BCS | -0.06 | 0.12 | -0.29 | 0.19 |
| λ_2_: Pw BCS | 0.23 | 0.22 | -0.21 | 0.65 |
| λ_3_: Gr BCS | -0.12 | 0.28 | -0.66 | 0.43 |
| λ_4_: ShGr BCS | 0.01 | 0.19 | -0.40 | 0.38 |
| λ_5_: NF BCS | 0.13 | 0.11 | -0.09 | 0.34 |
| λ_6_: Rf BCS | -0.01 | 0.50 | -1.02 | 0.91 |
| ψ: Time | -0.06 | 0.06 | -0.19 | 0.05 |
