# Supplementary material for: Towards evenly distributed grazing patterns: including social context in sheep management strategies
Source: PeerJ. 2016 Jun 21;4:e2152. doi: 10.7717/peerj.2152 (PMC4924134; doi:10.7717/peerj.2152)

Appendix S5: Space use of sheep

Figure S5.1: Trajectories duringtwo different days (a, b) of an ewe, a hogget and a wether at *Repunte Bajo* paddock under 3- category mixed flock management. In the trajectories of the upper panel (a) we can see that, even though hogget (blue) and ewe (orange) seemed to have a similar pattern of space use, they are seggregated when grazing on the wetlands. The hogget stayed on peripheral wetland (2) while ewe grazed on the central wetland (1). The movement path of the wether (black) was located mainly on the riparian and native forests (3). In the lower panel (b) we can see that the movement path of the ewe (orange) was more directed towards the central wetland (4), while the paths of the other two sheep was more distributes across the landscape. Patricularly, hoggets used more native forest and shrubland-grasland areas (5).


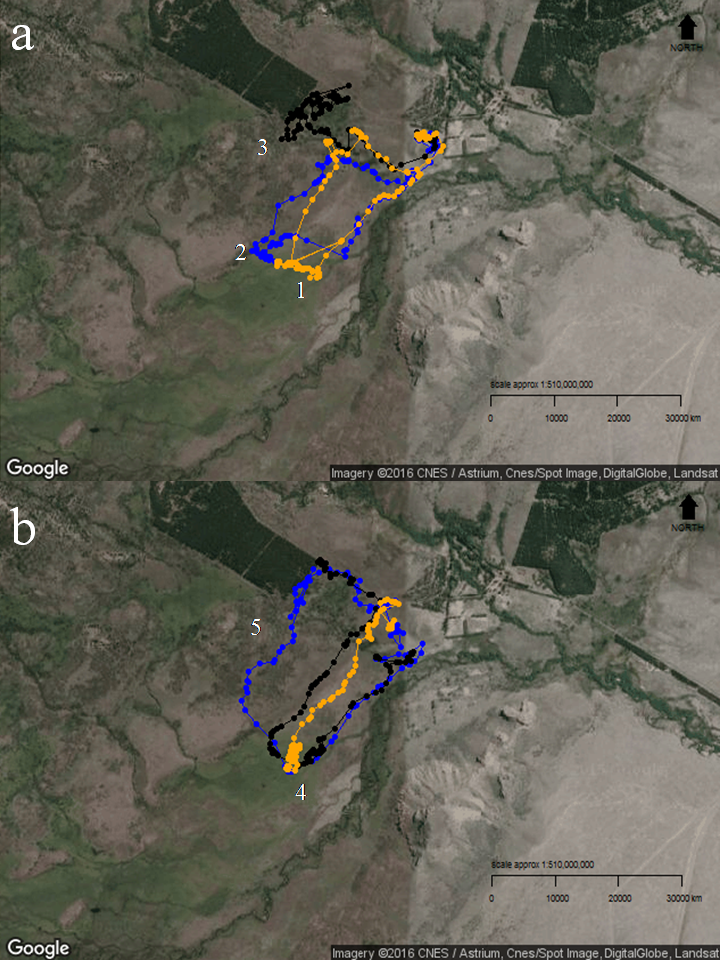


Figure S5.2: The upper panel (a) shows GPS locations of one hogget (blue), one ewe (orange) and a wether (black) grazing in a 3-category mixed flock during 30 days at *Repunte Bajo* paddock; and in the lower panel (b), shows GPS locations of one wether (black) and one hogget (blue) while grazing in a 2-category mixed flock during 30 days at the same paddock. We can see that when ewes were removed from the landscape (b), there were areas that remained completely under-grazed along the entire month, mainly those associated to riparian and native forests (1) and low production areas (2).


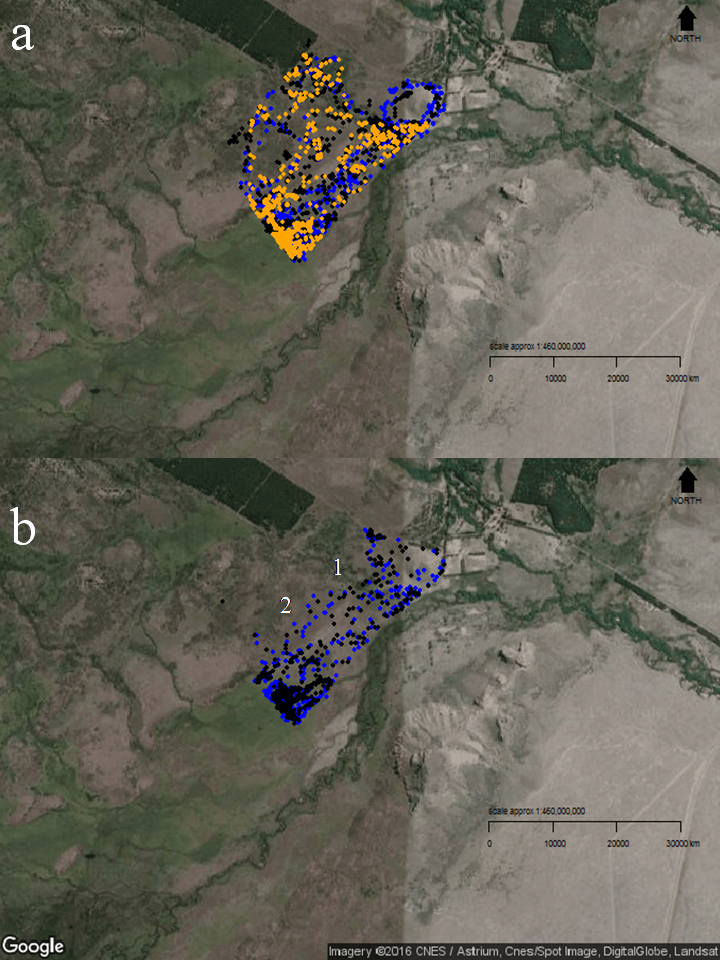


Figure S5.3: Trajectories during two different days (a, b) of a ewe, a hogget and a wether at *Side* paddock under 3- category mixed flock management. In the trajectories of panel (a) we can see that, the ewe (orange) showed a movement path almost entirely different than the hogget (blue) and the wether (black), using mainly peripheral wetland (1) and grassland (2). Meanwhile, the other two categories focused their activity on native forest and shrubland-grassland (3). The trajectories of panel (b) showed that ewe activity was more concentrated on the most profitable areas of the landscape: central (4) and peripheral (5) wetlands. On the other hand, hogget used native forest (6) and grassland (7).


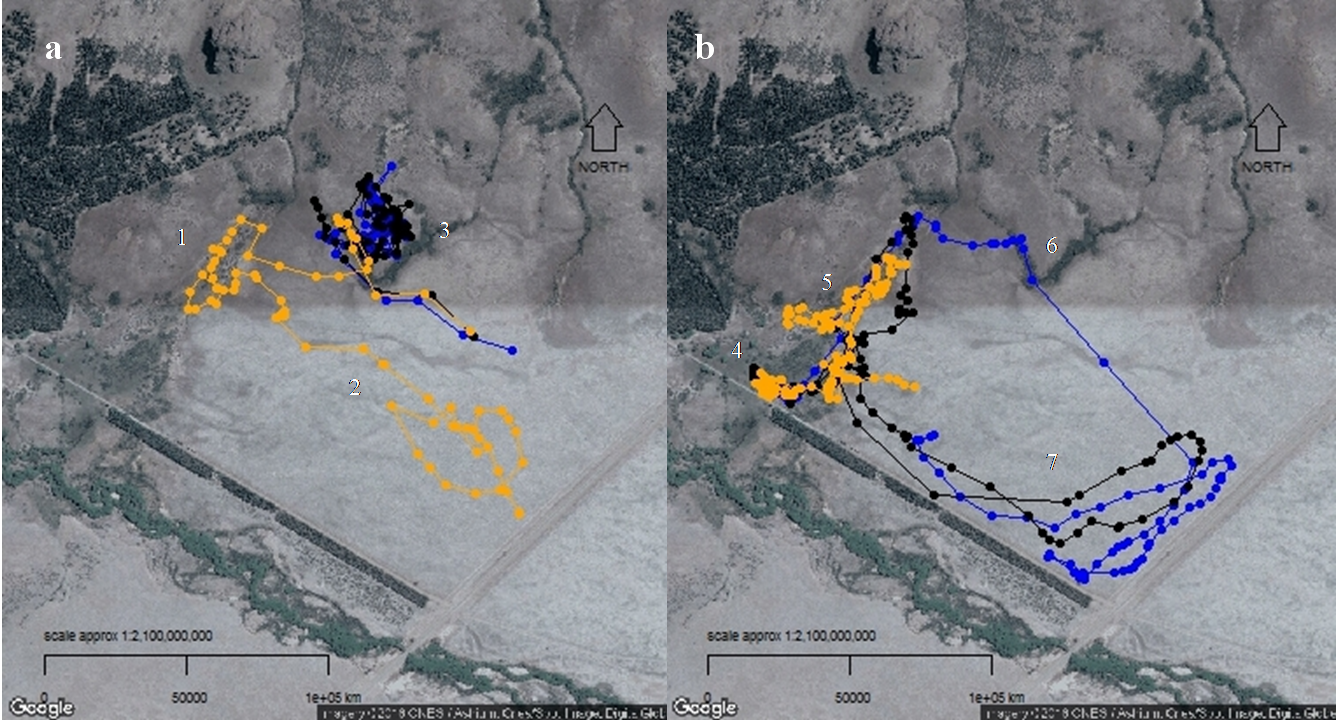


Figure S5.4: Trajectories during two different days (a, b) of a ewe, a hogget and a wether at *Frison-Guanaco* paddock under 3- category mixed flock management. We can see that for both days, the wether (black) and the ewe (orange) showed similar movement patterns, that differed from the hogget’s path (blue). During the first day presented here (a) ewe and wether used mainly highlands (1), while the hoggetused grassland (2) and concentrated its activity on native forest (3). During the second day, ewe and wether used highlands, grasslands and native forest, meanwhile, the hogget used grasslands (3) and an small wetland (4).


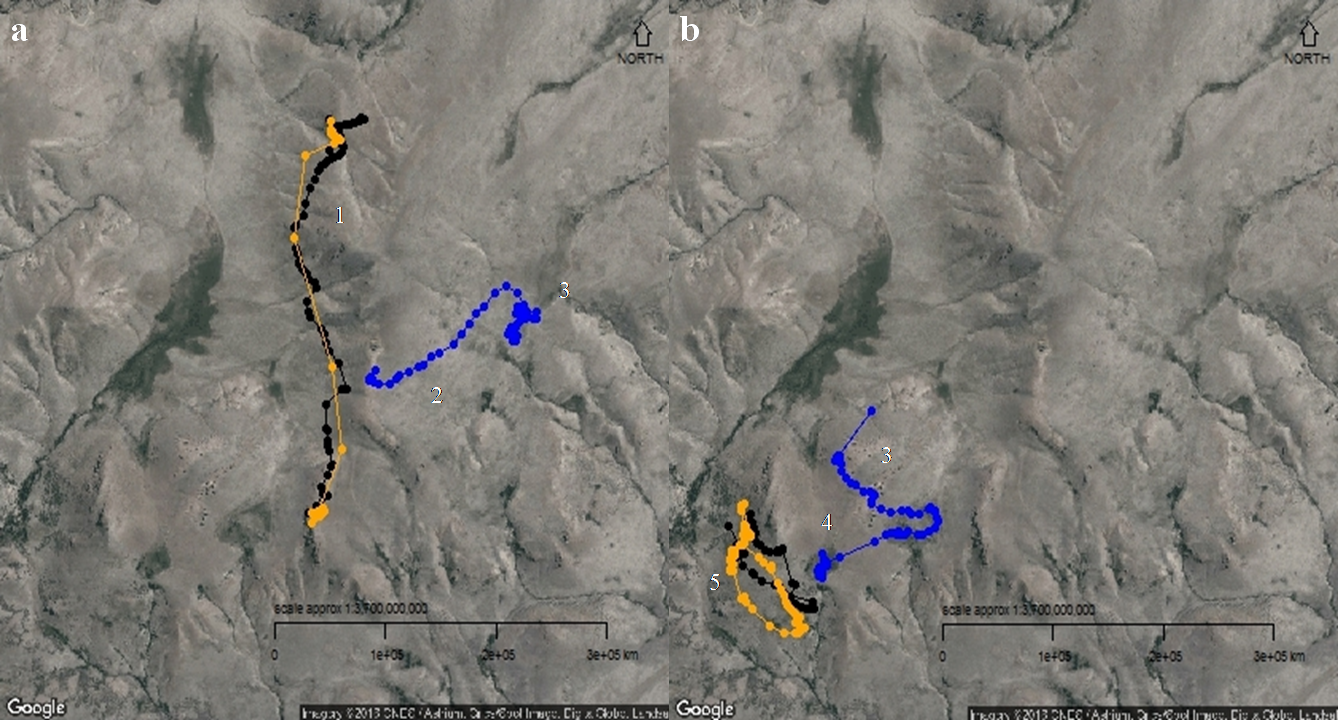

Supplement: Supplemental Information 5 [file peerj-04-2152-s005.docx]
